# Tea and *Pleurotus ostreatus* intercropping modulates structure of soil and root microbial communities

**DOI:** 10.1038/s41598-024-61883-w

**Published:** 2024-05-17

**Authors:** Zhengkai Yang, Jiaojiao Qu, Lu Qiao, Meiling Jiang, Xiao Zou, Wei Cao

**Affiliations:** 1 College of Tea Sciences, Guizhou University, Guiyang, 550025 China; 2 Institute of Fungus Resources, College of Life Sciences, Guizhou University, Guiyang, 550025 China; 3 Key Laboratory of Plant Resource Conservation and Germplasm Innovation in Mountainous Region (Ministry of Education), College of Life Sciences/Institute of Agro-Bioengineering, Guizhou University, Guiyang, 550025 China

**Keywords:** Tea, *Pleurotus ostreatus*, Intercropping, Microorganism, Chemical composition, Biolog® Eco-plates, Biological techniques, Ecology, Microbiology

## Abstract

Intercropping with *Pleurotus ostreatus* has been demonstrated to increase the tea yield and alleviate soil acidification in tea gardens. However, the underlying mechanisms remain elusive. Here, high-throughput sequencing and Biolog Eco analysis were performed to identify changes in the community structure and abundance of soil microorganisms in the *P. ostreatus* intercropped tea garden at different seasons (April and September). The results showed that the soil microbial diversity of rhizosphere decreased in April, while rhizosphere and non-rhizosphere soil microbial diversity increased in September in the *P. ostreatus* intercropped tea garden. The diversity of tea tree root microorganisms increased in both periods. In addition, the number of fungi associated with organic matter decomposition and nutrient cycling, such as *Penicillium*, *Trichoderma*, and *Trechispora*, was significantly higher in the intercropped group than in the control group. Intercropping with* P. ostreatus* increased the levels of total nitrogen (TN), total phosphorus (TP), and available phosphorus (AP) in the soil. It also improved the content of secondary metabolites, such as tea catechins, and polysaccharides in tea buds. Microbial network analysis showed that *Unclassified*_o__*Helotiales*, and *Devosia* were positively correlated with soil TN and pH, while *Lactobacillus*, *Acidothermus*, and *Monascus* were positively correlated with flavone, AE, and catechins in tea trees. In conclusion, intercropping with *P. ostreatus* can improve the physical and chemical properties of soil and the composition and structure of microbial communities in tea gardens, which has significant potential for application in monoculture tea gardens with acidic soils.

## Introduction

Tea (*Camellia sinensis* L.) is one of the most widely consumed non-alcoholic beverages in the world^1^. It is rich in polyphenols, which have potential health benefits related to reducing fatigue, detoxifying the body, quenching thirst, and improving vision^2,3^. Cultivated mainly in acidic red soil in subtropical and tropical regions, this plant is a significant perennial economic crop^4^. By 2023, China is the world’s leading tea producer, with a harvest area of 3.47 million hectares and producing 3.5 million tons of tea^5,6^. Nevertheless, while tea garden areas continue to expand yearly, there is an urgent need to tackle issues such as low average yield, enhancing tea quality, and improving the competitiveness of tea companies^7^.

The tea gardens in China have generally followed a monoculture model. However, the long-term monoculture of tea trees, due to their preference for acidic soils, can lead to aluminum (Al) accumulation, decreased soil pH, soil degradation, and a reduction in beneficial soil microbial communities^8,9^. In addition, as the market demand for tea products continues to grow, tea planters apply more fertilizer to increase tea production, causing a number of soil and environmental problems, such as nutrient leaching and an increased risk of heavy metal contamination. As a result, these factors can affect the soil microbial diversity and community structure, resulting in imbalances in the microbial community and reduced ecosystem service values of tea gardens^10^.

Microorganisms act as the driving force behind changes in soil ecosystems^11,12^. Their activities are responsible for nutrient cycling, decomposition of organic matter, soil structure, prevention of plant diseases, and an increase in plant productivity, all while being an integral part of soil functions^13^. Moreover, the soil physical and chemical characteristics are closely associated with the growth of plant roots and the uptake of nutrients^14^, as well as the composition of rhizosphere soil microbial communities^15^. The root exudates are known to attract beneficial microorganisms, thereby assisting the plant in its growth and in coping with environmental stress^16^. Therefore, the composition and variety of microbial communities can be used as indicators to assess soil health^12,17^. On that account, maintaining the diversity of soil microbial communities in tea gardens holds significant importance for managing soil nutrients and sustaining agricultural ecosystems.

Extensive research has shown that intercropping can be a useful way to improve tea plantations^18^. Compared to monoculture, interplanting with other crops (such as soybean and *Brassica napus*) in tea plantations can increase the soil pH^19^, raise soil organic carbon content^20^, and enhance the soil nitrogen, phosphorus, and potassium concentrations^18,21^. In addition, the levels of amino acids, sugars and oleic acid in fresh tea buds increased after intercropping, while the levels of bitter flavonoids decreased^22,23^. Apart from arable crops, intercropping with edible mushrooms has recently been practiced in different Chinese tea plantation systems. The edible mushroom species successfully intercropped in tea plantations include *Stropharia rugosoannulata*, *Morchella esculenta*, *Auricularia auricula*, *Ganoderma lucidum* and so on^24^. This intercropping plantation can have positive effects on tea gardens, including increasing tea yields, improving soil structure, reducing the risk of crop pests and diseases, and promoting the release and cycling of soil nutrients^25^. For example, intercropping *S. rugosoannulata* in a young tea garden during winter can bring forward the emergence of spring tea by 4.3 days and increase the tea bud density and quality per 100 buds by 15.1% and 8.0%, respectively^26^. Fungal species of *Basidiomycota*, *Ascomycota*, and *Mortierellomycota* significantly increased in the soil samples after intercropping with *Pleurotus ostreatus* in a tea garden^27^. In addition, interplanting edible mushrooms in tea gardens resulted in a significant increase in the abundance of beneficial microbial populations, such as *Burkholderia*, *Sphingomonas* and *Deinococcus* in the soil^28^.

*P. ostreatus* is an ideal species to intercrop with tea trees due to its rapid growth rate, simple cultivation process, and superior adaptation to the tea garden environment^29^*.* Our research has shown that intercropping *P. ostreatus* in tea gardens could lead to a marked improvement in the germination rate and yield of tea trees. Nevertheless, the precise effects of the fungi on soil chemical properties and microorganisms remain limited. In this study, the soil and tea trees in a tea garden, which were intercropped with *P. ostreatus* for four years, were used as materials. The following issues were explored: (1) the effects of intercropping *P. ostreatus* on soil chemical properties and tea quality in tea gardens, (2) how the diversity and composition of microbial communities changed under different intercropping seasons, and (3) the correlation of soil microbial community with soil chemical properties and tea quality. We hypothesized that intercropping with *P. ostreatus* could potentially change the species and abundance of native microorganisms in the early stages. Then the fungi may attract beneficial microorganisms such as *Bacillus*, *Azotobacter*, and *Trichoderma* to form a new network in the soil. This change may also affect the composition of endophytic microorganisms in the tea roots, resulting in a positive effect on the soil chemical properties and the tea quality.

## Materials and methods

### Materials

The experimental tea garden is located in Jiangkou County, Tongren City, Guizhou Province, China (24° 92' N; 114° 78' E). The annual average temperature is 16.2 °C, with an annual average precipitation of about 1,400 mm. Additionally, the annual sunshine hours are about 1,257 h, the altitude is approximately 400 m, and the frost-free period lasts for 294 days. The tea variety tested was "Fudingxiaoye", which was planted in 2011. The *P. ostreatus* strain was purchased from the Beijing Institute of Agricultural Resources and Regional Planning, Chinese Academy of Agricultural Sciences. The collection number was ACCC 52857, and the website was http://www.accc.org.cn/.

The collection of tea tree samples was carried out after obtaining permission from local suppliers. The authors confirm that this study complies with relevant legislation and international, national, and institutional guidelines.

### Experimental design

#### Substrate preparation, spawning, and culture of the *P. ostreatus*

The *P. ostreatus* strain (ACCC 52857) was cultured on Potato Dextrose Agar (PDA). The activating mycelia of *P. ostreatus* were inoculated into a 500 mL shaking flask with liquid PDA medium and cultured at 25 °C and 150 rpm for 5 days to prepare the initial liquid spawn. Then, the spawn was inoculated into a 50 L fermentor to prepare the second liquid spawn. The liquid medium consisted of the following components per liter: 35 g of glucose, 5 g of peptone, 4 g of yeast extract, 1 g of KH_2_PO_4_, and 0.5 g of MgSO_4_⋅7H_2_O^30^. For fruiting body production, the *P. ostreatus* strain was inoculated into solid media in polypropylene bags measuring 50 cm × 26 cm × 0.04 cm. The formula for solid media was as follows: 55% cottonseed hull, 30% sawdust, 10% bran, 3% gypsum, 0.5% potassium dihydrogen phosphate, 0.5% urea, and 1% glucose. The ratio of substrates to water was 1:1.5^31^. Each culture bag has a total weight of 4 kg and was sterilized at 100 °C for 7 h before being allowed to cool down to 25 °C. Each bag was then inoculated with 40 mL of the second liquid spawn. The bags were then incubated in a culture chamber under dark conditions at 23–25 °C for 25 days to promote the vegetative growth of *P. ostreatus* mycelia. It was continuously moistened to maintain relative humidity levels in the range of 80–90%, as described by Chang and Wasser^32^.

**Figure 1:**
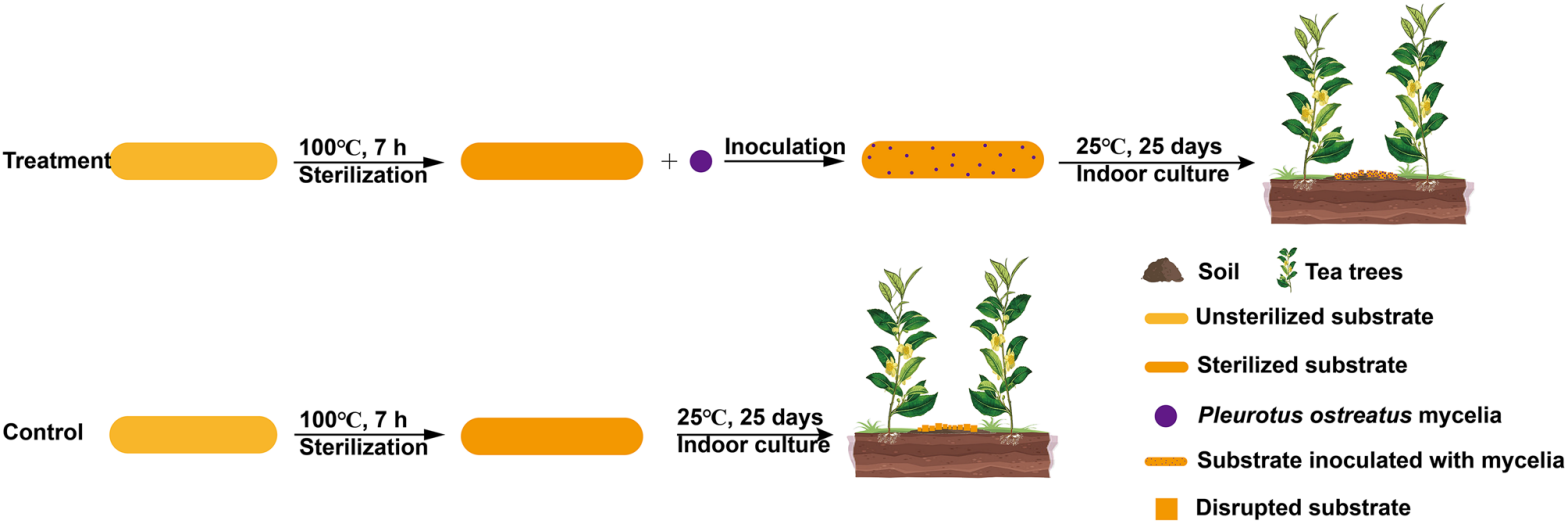
Experimental design diagram of *P. ostreatus* intercropping in a tea garden.

Once the substrate has been fully colonised, the next step is to intercrop the fungi on the wider side of the tea row for fructification. The first step was to remove the weeds from the surface of the tea garden. Then a trench was dug about 20 cm wide and 10 cm deep, making sure it was 10-15 cm from the stem of the tea plant to avoid damaging the main root system. The polypropylene membrane containing the bag of *P. ostreatus* mycelium was removed, and the substrate was crushed into particles 1–3 cm in diameter. The substrate was then laid flat on the bottom of the trench, approximately 2 cm thick, and covered with 2–3 cm of soil. The tea garden was irrigated continuously to ensure that the moisture retention rate of the fungal substrate was approximately 60%. The control group was received to the same sterile solid substrate but was not inoculated with *P. ostreatus* (Fig. 1). The intercropping took place from November to April of the following year, coinciding with the resting period of the tea garden. The intercropping model was implemented for three consecutive years (2019–2022).

### Sample collection

Soil and tea root samples were collected from the tea garden treatment area and the control area for the third year (including 36 samples collected in the spring and autumn of 2022). All samples were collected between 10:00 and 12:00 on April 1 and September 4, 2022. The soil and root samples included three different types: rhizosphere soil, non-rhizosphere soil, and tea root. The specific sampling scheme is shown in Supplementary Table S1. The sampling method was the five-point sampling method described by Jin et al.^33^. Five 0.3 m^2^ (5 tea plants) samples were randomly selected from each plot in the field of 30 m × 30 m field in the center of each group. The samples were then mixed and repeated three times. For non-rhizosphere soil samples, surface soil samples were taken at a distance of 10–20 cm from the tea stem at a depth of 25 cm, and the tea tree matrix was removed. After extraction of the soil samples, impurities such as dead branches and leaves were removed, and 10 g combination samples were extracted and used for sequencing soil bacteria and fungi. The remaining soil samples were used to assess their physical and chemical properties^34^.

To sample the rhizosphere soil and tea roots, the tea tree was selected using the five-point sampling method. The fine roots of five tea trees were collected from four different directions and mixed together. Then, the loose soil on the surface of the fine root and the stone between the roots were gently shaken off. The surface soil of the fine roots was then gently brushed off with a soft brush and collected as rhizosphere soil^35^. Each portion of approximately 10 g of soil was used to determine microbial metabolic diversity, and the remainder of the soil was used to determine physical and chemical indicators. To obtain endophytic microbial samples from tea plant roots, tea plant roots were collected using the method described above. At least 10 g of root samples were collected from each group. They were then disinfected with 75% (v / v) ethanol and 2% (v / v) NaClO for 2 min and 5 min, respectively. They were then rinsed 5–7 times with sterile water. Finally, the last rinsed sterile water was collected from the roots and then evenly distributed on the culture medium. If there are no bacteria or fungi in the medium, it can be concluded that the tea root has been effectively sterilized. After successful disinfection of the tea roots, they were divided into three parts and crushed with an appropriate amount of pre-cooled sterile 0.85% NaCl solution, which was used to analyze microbial metabolic diversity. Each group had three replicates.

For the sampling of tea buds, nine sampling points were randomly established for each treatment, and the area of each sampling point was 33.3 cm × 33.3 cm. All fresh tea buds (one bud and one or two leaves) within the sampling point area were collected, and the sum of fresh tea buds collected in each of the three plots was recorded as the density of tea buds per square meter^36^. The density of tea buds per square meter was calculated by counting one bud with one or two leaves of the tea tree in each sampling plot from these samples. The above samples were then mixed. One hundred bud leaves were randomly selected from the mixed samples and weighed. This process was repeated five times to measure the influence of the *P. ostreatus* intercropping mode on the tea garden^37^. Fresh tea bud samples were promptly transported back to the laboratory and stored at 4 °C for subsequent testing.

### Functional diversity study of microbial communities using Biolog® Eco-plates

A total of 10 g of samples collected in April (fresh soil and surface-sterilized tea tree roots) were placed in a conical flask containing 90 mL of sterilized normal saline (0.85% NaCl) and sealed with sterile cotton stoppers. The samples were shaken on a 180 rpm shaker for 30 min, followed by 15 min standing. Then 10 mL of the supernatant was extracted and diluted 1000 times with a 0.85% sterile solution. On an ultra-clean bench, 150 μL of the prepared soil and root microbial suspension was pipetted into each detection well of the Biolog® Eco plate (Biolog Inc, Hayward, CA, USA). The lid was secured with a rubber band and stored in a constant temperature incubator at 25 °C for 7 days. Absorbance readings were taken every 24 h at 18 °C and 590 nm using a microplate reader (Thermo Scientific, Waltham, MA, USA)^38^. To measure the total microbial activity, the average well color development (AWCD) was calculated for all carbon (C) sources. The following formula was used:

$${\text{AWCD }} = \, [\sum \left( {{\text{C}} - {\text{R}}} \right)]/{31}$$

where C is the optical density (OD) value of the soil inoculated wells, R is the OD value of the control well, and 31 is the number of carbon sources present in the Biolog® Eco-plates. The AWCD provides a comprehensive assessment of the utilization of different carbon sources by soil microbes^39^.

### The chemical composition of fresh tea shoots

#### Catechins and alkaloids content

The fresh tea bud sample was homogenized after being placed in a mortar filled with liquid nitrogen. The sample was weighed out to 0.200 g and placed in a 20 mL brown glass bottle. We then added 10 mL of a 20% methanol solution to the bottle and promptly placed it in the ultrasonic bath (25 kHz, 950 W, SCIENT2-IID) for 5 min. Filtration was conducted using a 0.45 μm filter membrane, and then 200 μL of the filtrate was transferred to a 2 mL brown chromatography vial. The chromatographic detection conditions were adopted from the Chinese national standards *GB/T 8313–2018*. Catechins, including catechin (C), catechin gallate (CG), gallocatechin (GC), gallocatechin gallate (GCG), epicatechin (EC), epigallocatechin (EGC), epicatechin gallate (ECG), epigallocatechin gallate (EGCG), caffeine (CAF), and gallic acid (GA) were analyzed. The samples were analyzed using high-performance liquid chromatography (HPLC) (UltiMate 3000, DIONEX, USA) equipped with a UV detector set at 278 nm. The analysis was performed on a C18 column (4.6 mm ID × 250 mm length, 5 μm particle diameter; Intersil ODS-3, Shimadzu, Japan) at a temperature of 35 °C. Mobile phase A consisted of a mixture of acetic acid (10 mg mL^−1^ EDTA-2Na) and water in a ratio of 1:500, while mobile phase B consisted of acetonitrile (HPLC grade). All eluents were filtered through a 0.45 μm filter membrane. The flow rate was 1 mL/min, and a 10 μL sample was injected into the column using gradient elution. The elution programme was as follows: 0–10 min, 0% of phase B; 10–25 min, 0–32% of phase B; 25–30 min, 32% of phase B; 30–35 min, 32–0% of phase B^36,40^.

### Polyphenols content

The Folin-Ciocalteu colorimetric method was used to determine the phenolic content of the tea leaf extracts according to the Chinese national standards *GB/T 8313-2018*. The Folin–Ciocalteu reagent was used to oxidize the tea leaf extracts, while sodium carbonate was used to stop the reaction. After incubation for 90 min at 25 °C, the absorbance of the samples was determined at 760 nm and then compared with the standards for known concentrations of gallic acid^41^.

### Theanine content

An automatic amino acid analyzer (Hitachi L-8900, Tokyo, Japan) was used to measure the leaf theanine content in the test tea buds^42^. Theanine content was determined by combining 5 mL of tea leaf extract with 5 mL of sulfo-salicylic acid and centrifuging the mixture at 13,000 rpm for 5 min, to promote the reaction. The mixture was filtered through a 0.22 μm filter membrane and analyzed using the amino acid analyzer.

### Soluble sugar content

The soluble sugar content in the tea buds was determined using the anthrone colorimetric method^43^. The basis for this determination is that when a concentrated sulfuric acid solution and sugar are combined, the resulting furfural or hydroxymethylfurfural can react with anthrone, leading to a change in the absorbance value at 630 nm.

### Aqueous extract and flavonoids content

Aqueous extract (AE) content was determined by the full weight method according to the guidelines of the Chinese national standards *GB/T8305-2013*^44^. Flavonoids were determined using the aluminum trichloride method as described by Zhang et al.^45^.

### Soil physicochemical analyses

After the soil was naturally air dried, the soil sample to be tested was filtered through a 0.45 mm sieve. The soil samples were mixed with deionized water at a ratio of 1: 2.5 (w/v), and the soil pH was measured using a pH meter (Lei-Ci, China) after standing for 30 min^46^. The soil organic carbon (SOC) content was determined using the potassium dichromate oxidation method. The heating instrument utilized the constant temperature oil bath external heating method (DingXinYi, China), and the absorbance value was measured using an absorbance photometer (Shimadzu, Japan)^47^. The total nitrogen (TN) and available nitrogen (AN) were analyzed through the Kjeldahl method, utilizing a Kjeldahl nitrogen analyzer supplied by Zhejiang Topu Yunnong Technology Co., Ltd^48^. After heating, the total phosphorus (TP), available potassium (AK) and total potassium (TK) of soil samples were analyzed using an atomic absorption spectrophotometer (Shimadzu, Japan) and heated in a box-type resistance furnace (Taisite, China)^47^. Soil available phosphorus (AP) was determined using the molybdenum antimony resistance colorimetric method, as described by Lu^47^.

### High-throughput sequencing

DNA extraction from soil and root samples was performed according to the Total DNA Extraction Kit (FastDNA® Spin Kit for Soil). Primers 799F (5'-AACMGGATTAGATACCCKG-3') and 1193R (5'-ACGTCATCCCCACCTTCC-3') were used to amplify the V5-V7 region of the bacterial 16S rRNA gene. The ITS region of the fungi was amplified with primers ITS1F (5 '-CTTGTCATTTAGAGAAGTAA-3') and ITS2R (5 '-GCTGCGTTTCTTCATCGATGC-3'). TransStart Fastpfu DNA Polymerase from TransGen AP221-02 was employed in the PCR process. PCR amplification was performed on an ABI GeneAmp & reg; Type 9700 instrument using 2 µL of DNA template, 2 µL of upstream and downstream primers (5 µmol/L), 4 µL of buffer (× 10), 2 µL of dNTP (2.5 mmol/L), 0.4 µL of FastPfu polymerase, 1 µL of sample DNA, and 8.6 µL of ddH_2_O. Reaction parameters as follows: 95 °C for 3 min; 25 × (95 °C 30 s; 45 °C 30 s; 72 °C 45 s); 72 °C for 10 min. The PCR products of the same sample were mixed and examined by a 2% agarose gel electrophoresis. The QuantiFluor™ -ST Blue fluorescence quantification system (Promega) was used to quantify the PCR products, and the initial electrophoresis quantification results were used as a reference. The samples were then mixed in proportions according to the sequencing volume requirements. The equimolecular weight amplifiers were purified and pooled on the Illumina MiSeq platform (Illumina, San Diego, CA, USA) according to the protocols of Majorbio Bio-Pharm Technology Co., Ltd. (Shanghai, China) standard protocol for paired-end sequencing (2 × 300 bp).

### Bioinformatic analyses

The paired-end (PE) reads obtained from MiSeq sequencing were aligned based on their overlap relationship, and the sequence quality was monitored and filtered. The effective sequences were obtained by distinguishing the sample and primer sequences at both ends of the sequence. The optimized sequence was obtained by correcting the direction of the sequence. OTU clustering was conducted on the QIIME platform. Sequences were grouped into operational taxonomic units (OTUs) using a 97% identity threshold. Fungal and bacterial OTUs were then compared against the UNITE and SILVA databases, respectively. The original data were stored in the NCBI Sequence Read Archive (SRA) database (SRA entry number: PRJNA985554; and the access link: http://www.ncbi.nlm.nih.gov/bioproject/985554).

UCHIME was used to identify and remove chimeric sequences. Rarefaction curves, Abundance-based Coverage Estimators (ACE), Chao1, Shannon–Wiener index and Simpson’s index were calculated and constructed based on operational taxonomic units (OTUs). Chao1 and ACE were calculated to estimate the abundance of fungal communities based on sequence differences. The Shannon–Wiener and Simpson indices were used to estimate the diversity within each sample^48^.

### Microbial network analysis

By constructing the microbial network of bacteria and fungi, the hub OTUs at the core of the corresponding microbial network were identified. The specific methods were as follows: first, the top 50 OTUs based on relative abundance in the sample were selected to construct a network. The correlation matrix of OTUs was calculated using the Spearman correlation method in the SPSS software package, and the p-value was adjusted using the False Discovery Rate (FDR). Among them, the network data was constructed based on the cloud platform of high-throughput sequencing, and the screening conditions were |r|> 0.8, *P* < 0.05. The network results were imported into Gephi software to calculate topological properties and visualize the network^49^. Finally, with reference to previous studies^50^, core microorganisms were screened based on the location of microorganisms in the network, the degree of passage of microorganism through nodes, proximity centrality, and betweenness centrality.

### Statistical analysis

First, SPSS 28.0 software package was used to analyze the significant differences in soil physical and chemical properties, chemical quality components, and fresh tea yield. Origin Pro 9.8 software (OriginLab, Northampton, MA, USA) was then used to generate histograms and other graphics. Duncan's Significant Difference test was used for post hoc statistical analysis, and permutation multivariate analysis of variance (PERMANOVA) was used to assess the significance of factors influencing the microbiome on soil physical and chemical properties, as well as the chemical composition quality of fresh tea buds. Non-metric multidimensional scaling (NMDS) was calculated using the Bray–Curtis dissimilarity to elucidate the relationship between all samples based on bacterial and fungal communities. The common and specific microbial OTUs of different samples were analyzed using a Venn diagram. The composition of the bacterial and fungal communities at the phylum and genus level was visualized and mapped using the Galluvial package. All tests were repeated three times, expressed as mean ± standard deviation (SD), and the significance level was set at *P* < 0.05.

### Ethics approval

This article does not contain any studies with human participants or animals performed by any of the authors.

## Results

### Effects of *P. ostreatus* intercropping on soil and tea root microbial metabolic activity (AWCD)

**Figure 2:**
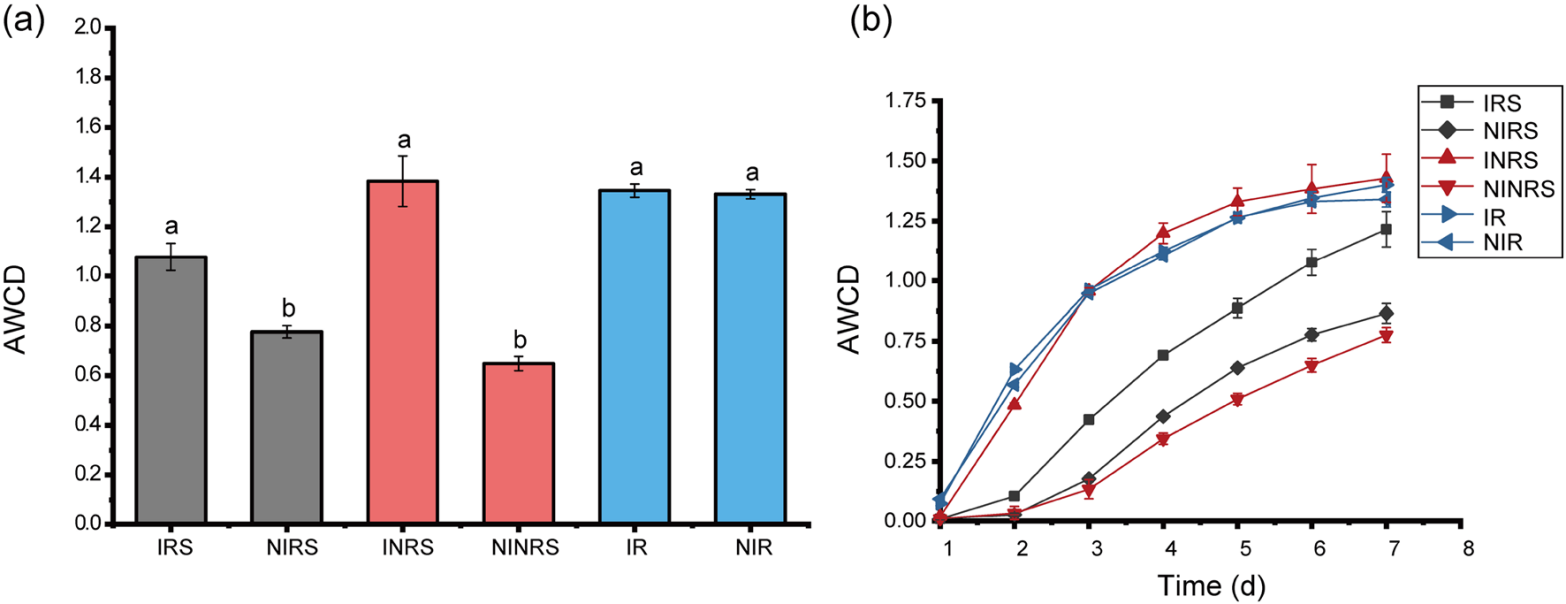
Effects of *P. ostreatus* intercropping on microbial carbon source utilization efficiency in soil and roots of a tea garden. (**a**) Histogram showing the efficiency of microbial carbon source utilization for different samples on the fifth day of culture, and (**b**) line graph showing the trend changes in the efficiency of microbial carbon source utilization for different samples.

The AWCD results indicated that the intercropping of *P ostreatus* had a positive effect on the microbial carbon source utilization efficiency of soils in tea gardens. The IRS (intercropped rhizosphere soil) and INRS (intercropped non-rhizosphere soil) increased by 38.6% and 113.1%, respectively (*P* < 0.05) compared to the control (NIRS, non-intercropped rhizosphere soil; NINRS, non-intercropped non-rhizosphere soil). However, there was no significant improvement in the root system (IR, the intercropped root) when compared to the control (NIR, the non-intercropped root) (Fig. 2a). From the change in carbon source utilization efficiency, INRS, IR, and NIR all exhibited high carbon source use efficiency, while NIRS and NINRS demonstrated low carbon source utilization efficiency. The IRS falls in the middle range (Fig. 2b).

### The yield and chemical composition of fresh tea shoots

The *P. ostreatus* was darker in color and denser in texture compared to those in the greenhouse (see Supplementary Fig. S1a). In addition, the roots of tea plants intercropped with *P. ostreatus* were more developed (refer to Supplementary Fig. S1d). This plant model can effectively promote the growth of tea buds (refer to Supplementary Fig. S1c). Compared with the control group (CK), the yield, bud weight, and density of the fresh tea shoots after intercropping with *P. ostreatus* showed a significant improvement in April (30.46%, 8.85%, 15.99%, respectively) and September (33.77%, 50.14%, 36.54%, respectively) (Supplementary Table S2, *P* < 0.05). In addition, the lateral of the tea tree roots in the intercropping line was denser (refer to Supplementary Fig. S1a,b).

The results from the biochemical component table showed that the levels of GC, C, and TB in the spring tea leaf samples were significantly higher than those in the September tea leaf samples for both treatment and control groups. The tea buds from the *P. ostreatus* intercropping group showed a significant increase in the levels of EGCG, EC, ECG, and polysaccharide in both April and September compared to the control group. The percentage increases were 262.95%, 196.52%, 119.47%, and 53.47%, respectively. Conversely, there were decreases of 44.44% and 9.28% in TB, and 25.29% and 15.11% in amino acids in both April and September compared to the control group. In April, the levels of CAF, tea polyphenols, and flavonoids decreased by 14.37%, 10.8%, and 28.14%, respectively. In September, these indices increased by 37.93%, 15.5%, 40.98%, and 23.06%, respectively. In the case of September, CG and GA experienced a decrease of 60.42% and 62.22%, respectively (refer to Supplementary Table S3). The results indicated that intercropping *P. ostreatus* in tea gardens had a direct effect on the biochemical quality of tea shoots, such as a significant increase in tea polysaccharides and catechins, which were positively correlated with the tea quality.

### Soil properties

According to Supplementary Table S4, intercropping with *P. ostreatus* significantly affected the soil properties of the tea garden. In April, the IRS and INRS groups showed significant increases in pH, total nitrogen (TN), total phosphorus (TP), total potassium (TK), and available phosphorus (AP) compared to the control group. The IRS group increased by 2.9%, 97.8%, 78.7%, 188.6%, and 31.2%, and the INRS group increased by 51.4%, 20.7%, 79.7%, and 78.8%, respectively. The IRS and INRS samples exhibited a notable decrease in soil organic carbon (SOC) and available nitrogen (AN) levels; the former decreased by 34.2% and 48.2%, while the latter decreased by 29.7% and 52.5%, respectively. In addition, the available potassium (AK) of the IRS experienced a significant decrease of 17.2%, while the INRS showed a significant increase of 186.1%. TN, TK, and AP of IRS and INRS increased in September by 11.9%, 66.5%, and 2.4%, respectively, while SOC decreased by 19.6% and 12.1% in IRS and INRS, respectively. The pH, TP, AN, and AK values of IRS decreased by 0.7%, 15.1%, 14.7%, and 19.1%, respectively, whereas the corresponding values of INRS increased by 13.7%, 3.7%, 32.5%, and 51.9% respectively. Intercropping with *P. ostreatus* increased the levels of TN, TK, and AP in the tea garden soil. In addition, NRS also increased the pH, TP, AN, and AK contents, while SOC content was lower than that in the control group.

### Microorganisms of soil and tea root

After quality control and removal of chloroplast or mitochondrial sequences, the OTU sequence similarity was 97%, and the classification confidence was 70%. A total of 1,440,028,542,681,868 bacterial bases and 1,701,043,405,568,712 fungal bases were detected in 18 samples collected in April. The bacterial species annotation results included 30 phyla, 76 classes, 193 orders, 372 families, and 822 genera. Fungal species annotation results included 13 phyla, 50 classes, 112 orders, 252 families, and 511 genera. At the same time, 18 samples were analyzed in September. A total of 1,600,346,602,706,671 bacterial bases and 1,684,671,396,515,350 fungal bases were detected. Bacteria belong to 30 phyla, 81 classes, 201 orders, 354 families, and 700 genera, while fungi belong to 13 phyla, 44 classes, 106 orders, 254 families, and 509 genera. The Shannon dilution curve of bacteria and fungi indicated that the bacterial diversity dilution curve gradually tended to saturate and stabilize at the 97% similarity classification level. This level could reflect the vast majority of the species information of the sample microorganisms (refer to Supplementary Fig. S2).

**Figure 3:**
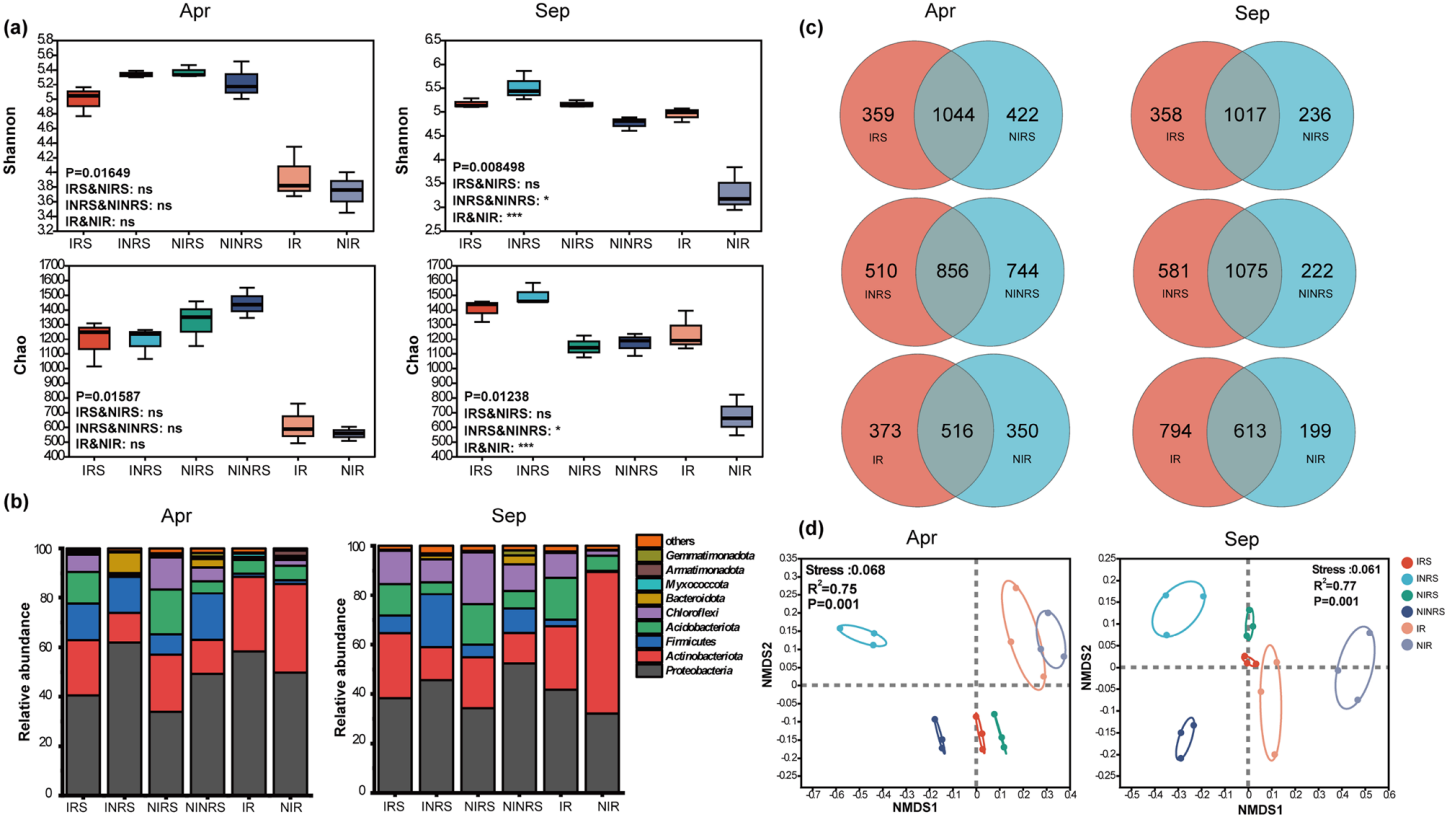
Composition and diversity of bacterial communities in the soil and roots of the tea garden. (**a**) Box plots of Shannon, Chao, Ace, and Simpson indices of bacterial communities in April and September, respectively. The line in the middle of the box plot represents the median, while the lines at both ends represent the minimum and maximum values. (**b**) Column diagram illustrating the community composition at the bacterial phyla level. (**c**) Venn diagram illustrating the bacterial microbial community in intercropped and non-intercropped tea plantations. (**d**) Non-metric Multidimensional Scaling (NMDS) diagram of bacterial communities in different samples.

**Figure 4:**
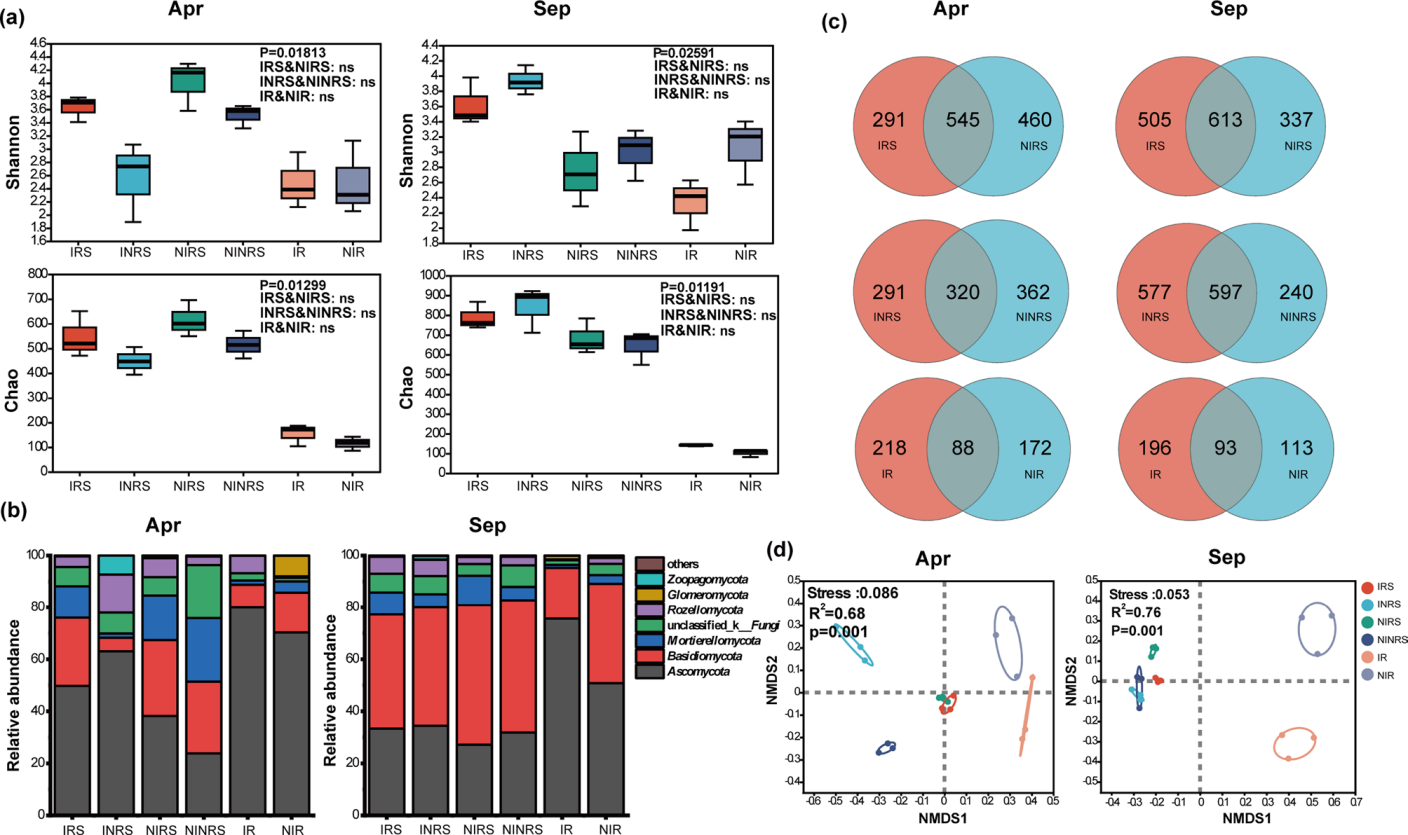
Composition and diversity of fungal communities in the soil and roots of a tea garden. (**a**) Box plots of Shannon, Chao, ACE, and Simpson indices of fungal microbial communities in April and September, respectively. The line in the middle of the box plot represents the median, while the lines at both ends represent the minimum and maximum values. (**b**) Column diagram illustrating the community composition at the fungal phylum level. (**c**) Venn diagram illustrating the fungal microbial community in intercropped and non-intercropped tea gardens. (**d**) Non-metric Multidimensional Scaling (NMDS) diagram of fungal communities in different samples.

In April, intercropping *P. ostreatus* reduced the diversity of IRS bacterial and fungal communities and increased the diversity of IR microbial communities. Whereas, the bacterial and fungal diversity of IRS, INRS, and IR increased in September (Figs. 3a, 4a; Supplementary Figs. S3 and S4). At the phylum level, the relative abundance of soil *Proteobacteria* and root bacteria was higher in intercropped tea gardens than in monocropped tea gardens, while the relative abundance of *Actinomycetes*, *Acidobacteria*, and *Chloroflexi* was lower than that of the control group in April. In September, the relative abundance of *Firmicutes* was higher than that of the control group, while the relative abundance of *Actinomycetes* in roots was lower than in the control group (Fig. 3b). At the genus level, the relative abundance of *Bacillus*, *Bradyrhizobium*, and *Burkholderia-caballeronia-para Burkholderia* in the intercropping soil and roots was lower than in the control tea garden in April. The relative abundance of *Acidothermus* in the rhizosphere soil of the intercropping tea garden was higher than that in the control tea garden, while the relative abundance of root *Acidothermus* was lower in the intercropping tea garden compared to the control tea garden. Whereas, the relative abundance of *unclassified_Thermosporaceae* in the roots of intercropped tea gardens was significantly lower than that of monocropped tea gardens, while the relative abundance of *unclassified_JG30-KF-AS9* was higher than that of monocropped tea gardens in September (refer to Supplementary Fig. S5).

In intercropped tea gardens, the fungal community showed significant changes in both the soil and root samples. In April, the relative abundance of *Ascomycota* increased while the relative abundance of *Basidiomycota*, *Mortierellomycota*, and *Glomeromycota* decreased. However, in September, the relative abundance of *Ascomycota* and *Zygomycota* increased while *Basidiomycota* and *Mortierellomycota* decreased (Fig. 4b). At the genus level, the relative abundance of *Mortierella* in intercropping soil and roots was lower than that in monoculture, while the relative abundance of *unclassified Helotiales* and *Trichoderma* in intercropping soil and roots was higher than that in monoculture in April. In September, the relative abundance of *Saitozyma* in the soil and roots of the intercropped tea garden after harvest was lower than that of the monoculture tea garden (see Supplementary Fig. S6).

Venn analysis showed that the bacterial and fungal microbial community in the tea garden was significantly affected after intercropping with *P. ostreatus*, resulting in substantial changes in both the bacterial and fungal community structures (refer to Figs. 3c and 4c). NMDS analysis based on Bray–Curtis distance revealed that the distribution of bacterial and fungal communities in the rhizosphere, non-rhizosphere soil, and roots of tea plants varied among different treatments. The distance between different processed samples is less than the distance between different types of samples. Therefore, the main reasons for the differences in microbial communities in tea gardens are the various types of samples, such as rhizosphere and non-rhizosphere (Figs. 3d and 4d).

### Microbial network analysis at different periods in tea gardens

**Figure 5:**
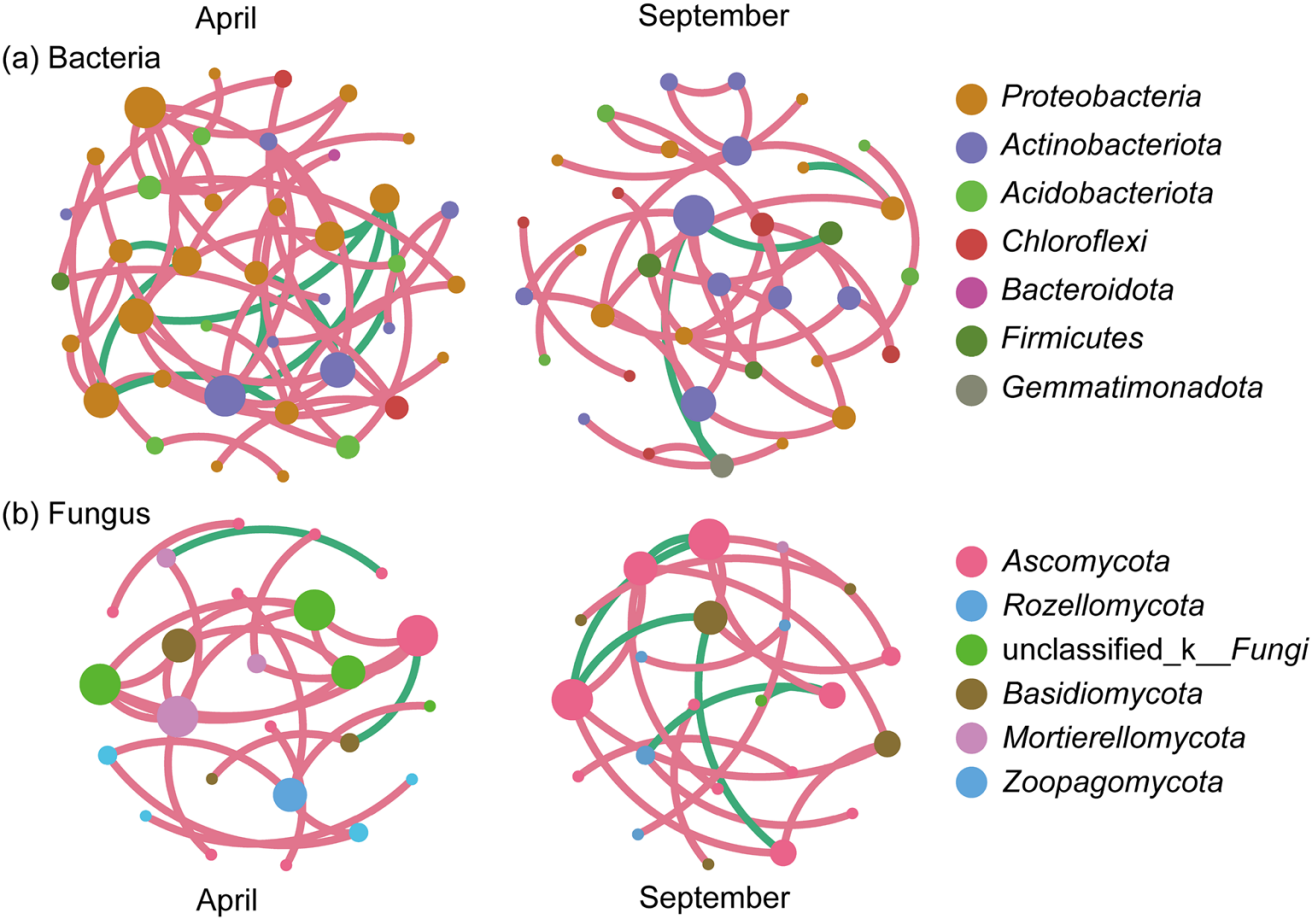
The co-occurrence networks based on OTU correlation analysis in the tea garden intercropped with *P. ostreatus* in April and September. (**a**) Bacterial and (**b**) fungal microbial correlation network.

The network analysis revealed that the number of nodes and edges, as well as the average degree and path distance of the microbial network in the tea garden, decreased in September compared to April. Therefore, in September, the complexity of fungal and bacterial networks decreased (Fig. 5 and Supplementary Table S5). By screening the species with a node degree greater than four, a closeness centrality greater than 0.4, and a betweenness greater than 0.3 in the network, we identified them as the hub species, which were shown in Supplementary Tables S6 and S7. The results showed that there were eight core species in the bacterial network, such as *Bradyrhizobium*, *Acidothermus*, and unclassified_f__*Thermomonospora ceae*, and seven core species in the fungal network, such as *Mortierella*, *Oidiodendron*, and *Apiotrichum* in April. In September, the bacterial network had two central species, such as *Acidothermus*, while the fungal networks had four core species, such as *Trichoderma* and *Thermoascus*.

### The relationship between soil properties and microbial communities

**Figure 6:**
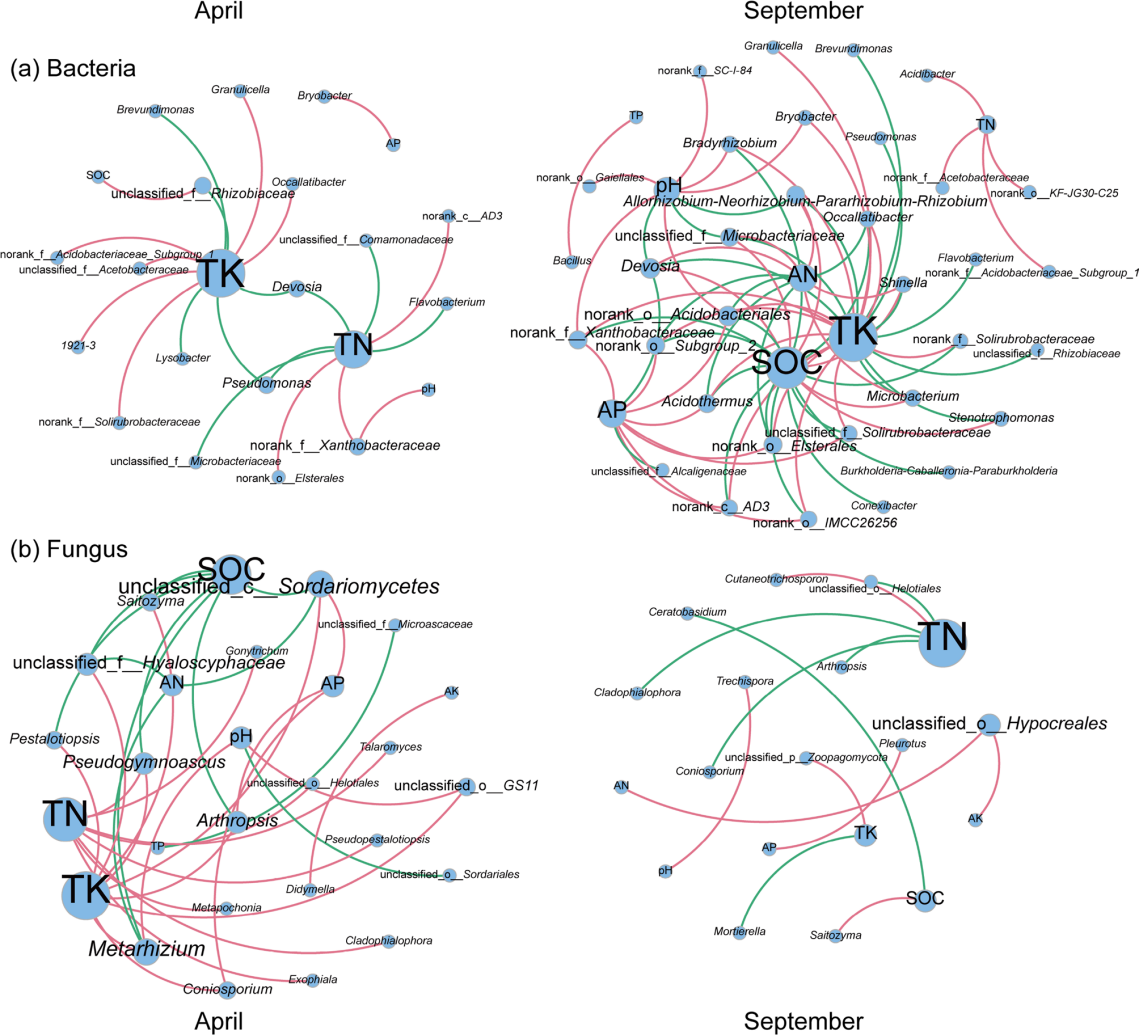
Correlation network analysis of soil physical and chemical properties with (**a**) bacterial and (**b**) fungal communities in tea garden intercropped with* P. ostreatus.*

Correlation network analysis of the soil microbial community and soil physical and chemical properties in a tea garden showed that, compared to April, the average number of nodes, edges, and average degree of the bacterial microbial network increased in September, while the average number of nodes, edges, and average degree of the fungal microbial network decreased. This implies that the bacterial network is more complicated, while the fungal network is simpler (Fig. 6 and Supplementary Table S8). SOC, TK, and AP showed significant correlations with the microbial community. The results of the PERMANOVA analysis indicated significant differences between SOC, TK, and AP and the microbial communities in two different seasons (refer to Supplementary Table S9, *P* < 0.01). The microbial communities exhibited the strongest correlation with TK. In addition, core microorganisms associated with soil physical and chemical properties were screened (see Supplementary Table S10), and 14 bacterial and five fungal core microorganisms were identified during two sampling periods (April and September). The top five core bacteria included *Norank_f_Xanthobacteraceae, Norank_c_AD3, Norank_f_Acidobacteraceae_Subgroup_1, Granulicella,* and *Devosia*. The top five fungal core microorganisms were unclassified *Helotiales*, *Saitozyma*, *Coniosporium*, *Cladophora*, *and Arthropsis*.

### The correlations between tea chemical composition and microbial communities

**Figure 7:**
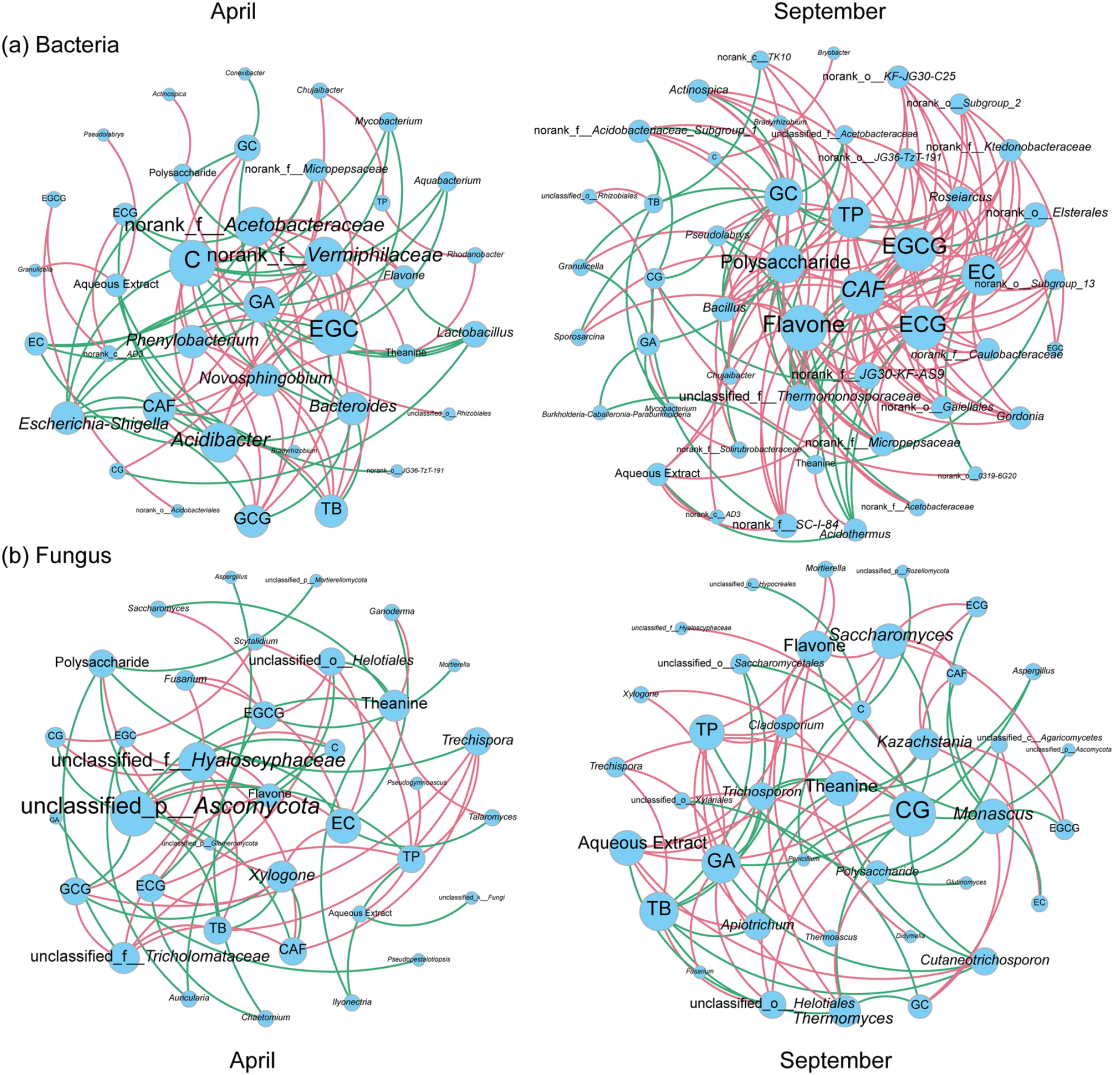
Correlation network analysis of tea chemical properties with the bacterial (**a**) and fungal (**b**) communities in a *P. ostreatus* intercropped tea garden.

In September, a correlation network analysis of the tea rhizosphere microbial community and tea chemical characteristics revealed that the number of nodes, edges, and average degree of the microbial network increased and the average path length decreased compared to April. Moreover, the bacterial network was more complex than the fungal network (Fig. 7 and Supplementary Table S11). The core microorganisms of the tea rhizosphere microbial community in the network related to tea chemical characteristics were identified (Supplementary Table S12). In both the April and September samples, there were seven core microorganisms, including bacteria and fungi. According to the network connectivity, the top three bacteria were *Actinospica, Norank_f_Acetobacteraceae* and *Micropepsaceae.* The top three fungi were *unclassified_Ascomycota, unclassified_Helotiales* and *unclassified_Hyalomycetaceae.* PERMANOVA analysis in April and September showed that AE, theanine, and TP had the most significant effects on the root microbial community (refer to Supplementary Table S13; *P* < 0.01).

## Discussion

Our research has shown that intercropping with *P. ostreatus* in a tea garden has a notable effect on both spring and autumn bud germination, but a more remarkable effect on spring tea growth compared to the monoculture garden. In this study, high-throughput sequencing and Biolog Eco coupled with were utilized to analyze these changes from a microbial perspective. Throughout the intercropping period (from November to April of the following year), there was an initial decrease in the diversity of soil microbial communities, followed by an increase in September. It is possible that the *P. ostreatus* thrives in the tea garden by rapidly adapting and then outcompeting the native soil microorganisms. After the mushroom harvest (May to September), the remaining mycelia and substrate continue to remain in the tea line. The organic matter in the substrate decomposes more completely than in the control group, allowing it to maintain nutrients for microorganisms in the soil. As a result, the diversity of rhizosphere microbial communities increased significantly 5 months after the mushroom harvesting. Within a certain range, changes in the soil microbial community in a tea garden then affect the yield and quality of tea trees.

Previous studies have shown that intercropping soybeans, walnuts, and white beans in tea gardens can improve tea quality by enhancing soil nutrients and reducing heavy metal content. However, the investigation of the mechanism linking tea quality with rhizosphere soil and root microorganisms remains limited. In this study, we found that intercropping *P. ostreatus* with tea trees can improve the physical and chemical properties of the soil, such as increasing the soil pH, TN and TK. Moreover, the indices of physical and chemical properties in rhizosphere soil were better in April than in September, compared to the control group. Interplanting *P. ostreatus* among tea garden can improve the physical and chemical properties of the soil, which was confirmed by the study of Ma et al.^27^. However, the changes in soil properties in tea gardens following harvesting of *P. ostreatus* have not been investigated. In this study, significant changes were observed in the physical and chemical properties of both rhizosphere and non-rhizosphere soil following harvesting. The physical and chemical properties of the rhizosphere soil decreased significantly after harvesting the mushrooms, while those of the non-rhizosphere soil increased significantly. This phenomenon may be attributed to the legacy effect of soil microorganisms^51^. Soil acidification in tea gardens is an inevitable issue. pH is considered a predictor of microbial community diversity in soil, with higher pH correlating with greater alpha diversity of microbial populations within a specific range^52^. This phenomenon may be attributed to the enhanced adaptability of most microbial cells to a neutral pH environment^53^. We observed that the soil pH remained stable despite the rise in alpha diversity of the non-rhizosphere soil microbial community in an intercropped tea garden in April.

In addition, this study found that the microbial community of rhizosphere soil and non-rhizosphere soils significantly increased the utilization efficiency of carbon sources in intercropped soil. However, the microbial community of the non-rhizosphere soil exhibited a greater increase. This may be caused by the rapid growth of *P. ostreatus* mycelium competing for ecological niches with native microorganisms in the rhizosphere soil, leading to a reduction in the diversity and abundance of other native microorganisms. Increasing the utilization efficiency of carbon sources leads to increased decomposition of organic matter in the tea garden. This can help to maintain the necessary nutrients for the growth of micro-organisms in the tea trees and soil^54^. Interestingly, we observed that the alpha diversity of microbial communities in intercropping tea roots notably increased in April and September compared to the control group. It suggests that intercropping *P. ostreatus* influenced the diversity of microorganisms that could be transferred into the tea roots, and then enhanced the diversity of microbial communities in the tea roots.

The intercropping with *P. ostreatus* can slightly reduce the diversity of bacterial communities while protecting competitive fungi^55^. The intercropping of *P. ostreatus* reduced the proportion of *Acidothermus* in the soil, which is usually considered to produce cellulase^56^. Therefore, the decrease in the proportion of *Acidothermus* may be attributed to its competitive relationship with *P. ostreatus*^57^. Consequently, the relative abundance of *Trichoderma* increased in this competitive relationship, suggesting that *Trichoderma* could serve as an alternative to *Acidothermus*. Unclassified *Helotiales* is a mycorrhizal fungus whose main function is thought to transport nutrients, mainly nitrogen, to plants. It was positively correlated with the total nitrogen (TN) of the soil. *Devosia*, a common rhizobium, was positively correlated with total nitrogen (TN) and pH^58^. In the soil intercropped with *P. ostreatus*, the abundance of both of the above bacteria was significantly increased, which may be one reason for the rise in soil pH and total nitrogen (TN) content. *Monascus* is used to ferment Pu'er tea and contributes to its distinctive aroma^59^. This study found that *Lactobacillus*, *Acidothermus*, and *Monascus* were positively correlated with flavone, AE, CAF, TB, and catechins. Particularly, *Monascus* was positively correlated with EGCG. Therefore, these results suggest that *Lactobacillus*, *Acidothermus*, and *Monascus* may be the key microorganisms in soil that are positively associated with the tea quality.

To further explore changes in the ecological function of the microbiota caused by intercropping with *P. ostreatus*, we carried out functional prediction analyses of the fungal and bacterial communities for different samples. It was found that the changes in microbial function occurred mainly in the soil fungal community. In April, the treatment group exhibited a higher abundance of undefined saprotrophs and the endophyte-litter saprotroph-soil saprotroph-undefined saprotrophs, which play a key role in decomposition and nutrient cycling in soil ecosystems. By September, wood saprotrophic micro-organisms dominated the treatment group, while undefined saprotrophic fungal parasites predominated in the control group (Supplementary Fig. S7). In addition, the FUNGuild analysis was used to screen the microorganisms associated with wood saprotroph and undefined saprotroph (Supplementary Table S14). *Penicillium* and *Trichoderma* were found to be associated with undefined saprotroph, while *Trechispora* was associated with wood saprotroph. These saprotrophs can decompose complex molecules such as cellulose and other nonliving organic matter, potentially increasing nutrient provision compared to the control tea garden.

In conclusion, intercropping with *P. ostreatus* in winter tea gardens is an economical and effective way of increasing tea garden production. However, more work needs to be done on the science and rationale of this planting pattern. For example, further experiments are required to confirm the specific functions of these microorganisms. Using culturable isolation methods, these strains can be obtained and then used to construct artificial microbial communities through different combinations of strain functions. By inoculating the artificial community with tea tree, the interactions between the community and the tea tree will be studied.

## Conclusion

In this study, we found that intercropping *P. ostreatus* in a tea garden could significantly increase the tea yield and improve the soil physical and chemical properties, such as pH and TN content. In addition, Biolog Eco analysis showed that intercropping *P. ostreatus* could significantly improve the utilization efficiency of microbial carbon sources in the soil. High-throughput sequencing analysis further validated that the diversity of microbial communities increased and the microbial community structures were improved in September when intercropped with *P. ostreatus*. The number of fungi associated with organic matter decomposition and nutrient cycling, such as *Penicillium*, *Trichoderma* and *Trechispora*, was significantly higher in the intercropped group than in the control group, which may improve the nutrient availability of the tea plantation soil and promote tea tree growth. The genus *unclassified_o__Helotiales* and *Devosia* may be the key microorganisms responsible for TN and pH changes in soil, while *Lactobacillus*, *Acidothermus* and *Monascus* have a closer relationship with tea quality.

### Supplementary Information

Supplementary Information.

## Data Availability

The datasets mentioned in this study are available upon request from the corresponding authors. Please contact us at jjqu@gzu.edu.cn.
