# Supplementary material for: Tea and Pleurotus ostreatus intercropping modulates structure of soil and root microbial communities
Source: Sci Rep. 2024 May 17;14:11295. doi: 10.1038/s41598-024-61883-w (PMC11101613; doi:10.1038/s41598-024-61883-w)
Supplement: Supplementary file 1 — Supplementary Information. [file 41598_2024_61883_MOESM1_ESM.pdf]

Article title: Tea and *Pleurotus ostreatus* intercropping modulates structure of soil and root microbial communities

Author names: Zhengkai Yang, Jiaojiao Qu*, Lu Qiao, Meiling Jiang, Xiao Zou, Wei Cao*

Affiliation and E-mail address of corresponding author:

College of Tea Sciences, Guizhou University, Guiyang, 550025, China; jjqu@gzu.edu.cn (J. Q.); Institute of Fungus Resources, College of Life Sciences, Guizhou University, Guiyang, 550025, China; gdwcao@163.com (W. C.)

Supplementary tables

Table S1 The sampling design

Group	Sample type	Non-intercropped group	Intercropped group
Spring (April 2022)	Non-rhizosphere soil	1×3	1×3
	Rhizosphere soil	1×3	1×3
	Tea tree root	1×3	1×3
Autumn (September 2022)	Non-rhizosphere soil	1×3	1×3
	Rhizosphere soil	1×3	1×3
	Tea tree root	1×3	1×3

Table S2 Tea yield of tea garden in different seasons. Apr Represent April, Sep Represent September, the same below.

		Yield of tea leaves (kg/hm^2)	100 - sprout weight (g)	Sprout density ($number/m^2$)
Apr	IP	1102.08±3.717a	29.52±0.377a	1136.82±9.900a
	CK	844.78±3.972b	27.12±0.272b	980.10±17.541b
Sep	IP	246.04±0.581a	15.72±1.600a	521.58±22.800a
	CK	183.93±0.367b	10.47±0.793b	382±21.803b

Table S3 Chemical quality components of tea shoots in different seasons. IP means intercropping with P. ostreatus and CK means control group; GC, EGC, C, EGCG, EC, GCG, ECG, CG, GA, TB, CAF and AE represent gallocatechin, epigallocatechin, catechin, epigallocatechin gallate, epicatechin, gallatechin gallate, epigallocatechin gallate, catechin gallate, gallic acid, theobromine, caffeine and aqueous extracts, respectively. Different letters in the same column indicate a significant difference ($P<0.05$).

	Apr		Sep	
Sample	IP	CK	IP	CK
GC	23.99±1.050a	24.30±1.301a	3.99±2.098a	10.49±0.349a
EGC	0.40±0.023a	0.46±0.023a	0.80±0.118a	0.71±0.034a
C	10.17±0.384a	11.78±0.609a	2.23±0.005a	2.01±0.190a
EGCG	199.31±3.881a	54.19±0.312b	192.08±13.247a	61.59±1.690b
EC	6.82±0.037a	2.30±0.032b	6.71±0.685a	2.23±0.074b
GCG	2.75±0.119a	3.34±0.188a	1.14±0.139a	0.92±0.025a
ECG	39.00±0.204a	17.77±0.119b	38.50±2.068a	17.10±0.534b
CG	0.53±0.009a	0.56±0.024a	0.19±0.011b	0.48±0.014a
GA	6.40±0.296a	6.62±0.362a	1.67±0.072b	4.42±0.144a
TB	1.05±0.040b	1.89±0.086a	0.65±0.046b	0.87±0.032a
CAF	51.29±1.604b	59.90±2.281a	44.04±1.914a	31.93±0.972b
AE	359.14±4.698a	361.22±1.468a	427.17±4.287a	369.84±6.282b
Theanine	17.31±0.249b	19.08±0.151a	15.45±0.797b	18.20±0.373a
Polyphenols	93.26±0.649b	104.55±1.988a	133.13±3.934a	94.43±0.718b
Polysaccharide	156.42±0.286a	101.92±1.379b	198.95±1.388a	150.20±3.001b
Flavonoids	18.59±0.298b	25.87±0.737a	26.31±0.269a	21.38±0.412b

Table S4 Soil properties of tea garden in different seasons. pH, SOC, TN, TP, TK, AN, AP and AK represent soil pondus hydrogenii, soil organic carbon, total nitrogen, total phosphorus, total potassium, available nitrogen, available phosphorus and, available potassium, respectively. There is a significant difference between different letters in the same line (*P*<0.05).

		IRS	NIRS	INRS	NINRS
Apr	pH	4.24±0.09b	4.12±0.410c	5.59±0.041a	4.26±0.013b
	SOC(g/kg)	1.21±0.055c	1.79±0.072a	0.73±0.069d	1.41±0.024b
	TN(g/kg)	0.89±0.072a	0.45±0.063bc	0.56±0.030b	0.37±0.030c
	TP(g/kg)	0.58±0.006a	0.38±0.001b	0.35±0.007c	0.29±0.016d
	TK(g/kg)	34.03±0.245b	19.04±0.068d	36.44±0.255a	20.28±0.230c
	AN(mg/kg)	47.41±1.686b	67.37±1.361a	23.89±0.887c	50.25±2.367b
	AP(mg/kg)	2.54±0.031b	0.88±4.031c	5.77±0.031a	0.85±0.031c
	AK(mg/kg)	213.45±7.735c	257.71±4.158b	756.75±3.482a	264.51±13.685b
Sep	pH	4.52±0.003c	4.55±0.003b	5.06±0.009a	4.45±0.006d
	SOC(g/kg)	2.42±0.040b	3.01±0.158a	2.18±0.021b	2.48±0.275b
	TN(g/kg)	0.75±0.026b	0.67±0.006bc	0.87±0.048a	0.60±0.042c
	TP(g/kg)	0.45±0.004c	0.53±0.006b	0.83±0.010a	0.80±0.014a
	TK(g/kg)	27.79±0.042b	16.69±0.189d	30.10±0.295a	18.10±0.232c
	AN(mg/kg)	53.06±5.063d	62.18±5.428c	191.93±3.967a	144.81±4.628b
	AP(mg/kg)	2.56±0.031c	2.50±0.031c	11.48±0.031a	8.10±0.031b
	AK(mg/kg)	191.74±5.704d	237.05±3.772c	664.81±9.545a	437.70±12.134b

Table S5 Co-occurrence network of core microorganisms in tea garden at different periods

		average degree	Average clustering coefficient	average path length
bacteria	April	2.462	0.499	2.456
	September	2.171	0.659	2.144
fungus	April	2.25	0.486	2.451
	September	2	0.441	2.338

Table. S6 Core microorganism of bacterial co-occurrence network

	Id	Phylum	Genus	Degree	Closeness Centrality	BetweennessCentrality
April	OTU2068	*Proteobacteria*	*Bradyrhizobium*	6	0.55	35.5
	OTU3190	*Actinobacteriota*	*Acidothermus*	6	0.55	17.33
	OTU2441	*Actinobacteriota*	*unclassified_f__Thermomonosporaceae*	5	0.52	9
	OTU2221	*Proteobacteria*	*Bradyrhizobium*	5	0.6	20.33
	OTU2314	*Proteobacteria*	*Devosia*	5	0.57	46
	OTU2139	*Proteobacteria*	*norank_f__norank_o__KF-JG30-C25*	4	0.48	29
	OTU2408	*Proteobacteria*	*Acidibacter*	4	0.57	10
	OTU892	*Proteobacteria*	*unclassified_f__Alcaligenaceae*	4	0.48	0.33
September	OTU2001	*Actinobacteriota*	*unclassified_f__Thermomonosporaceae*	6	0.67	28
	OTU1815	*Actinobacteriota*	*Acidothermus*	5	0.53	4

Table. S7 Core microorganism of fungal co-occurrence network network

	Id	Phylum	Genus	Degree	Closeness Centrality	BetweennessCentrality
April	OTU866	*Mortierellomycota*	*Mortierella*	5	0.48	29
	OTU1115	*unclassified_k__Fungi*	*unclassified_k__Fungi*	5	0.55	13
	OTU989	*Ascomycota*	*Oidiodendron*	5	0.52	36
	OTU1230	*unclassified_k__Fungi*	*unclassified_k__Fungi*	5	0.55	13
	OTU1159	*Basidiomycota*	*Apiotrichum*	4	0.44	1.33
	OTU368	*Zoopagomycota*	*unclassified_p__Zoopagomycota*	4	1	5
	OTU1218	*unclassified_k__Fungi*	*unclassified_k__Fungi*	4	0.46	1.67
September	OTU1462	*Ascomycota*	*Trichoderma*	5	0.52	8
	OTU1907	*Ascomycota*	*Thermoascus*	5	0.61	32
	OTU440	*Ascomycota*	*Trichoderma*	4	0.5	12.75
	OTU2383	*Basidiomycota*	*Trichosporon*	4	0.48	4.5

Table S8 Soil physical and chemical properties and soil microbial co-occurrence network in tea garden

		node	edge	average degree	Average clustering coefficient	average path length
bacteria	April	23	22	1.913	0	2.81
	September	40	76	3.8	0	2.232
fungus	April	27	35	2.593	0	2.949
	September	19	13	1.368	0	1.5

Table S9 PERMANOVA between soils and microbes

	Bacterial				Fungi			
	Apr		Sep		Apr		Sep	
	R^2	*P*	R^2	*P*	R^2	*P*	R^2	*P*
SM	0.831	0.001	0.745	0.001	0.72	0.001	0.62	0.001
SOC	0.422	0.001	0.416	0.002	0.315	0.001	0.324	0.001
TK	0.436	0.001	0.291	0.003	0.359	0.001	0.306	0.001
AP	0.346	0.001	0.251	0.016	0.257	0.006	0.284	0.002
AN	0.406	0.002	0.231	0.025	0.295	0.007	0.34	0.005
pH	0.259	0.005	0.149	0.153	0.217	0.014	0.266	0.005
AK	0.209	0.045	0.14	0.163	0.179	0.041	0.268	0.008
TN	0.204	0.054	0.137	0.173	0.179	0.047	0.252	0.011
TP	0.139	0.196	0.135	0.203	0.14	0.141	0.185	0.052

Table S10 Physical and chemical properties of soil and core microorganism of soil microbial co-occurrence network

Bacteria		Fungus	
Phylum	Genus	Phylum	Genus
Proteobacteria	*unclassified_f__Rhizobiaceae*	*Ascomycota*	*unclassified_o__Helotiales*
Actinobacteriota	*unclassified_f__Microbacteriaceae*	*Basidiomycota*	*Saitozyma*
Proteobacteria	*Pseudomonas*	*Ascomycota*	*Coniosporium*
Acidobacteriota	*Occallatibacter*	*Ascomycota*	*Cladophialophora*
Proteobacteria	*norank_f__Xanthobacteraceae*	*Ascomycota*	*Arthropsis*
Actinobacteriota	*norank_f__Solirubrobacteraceae*		
Chloroflexi	*norank_c__AD3*		
Proteobacteria	*norank_o__Elsterales*		
Acidobacteriota	*norank_f__Acidobacteriaceae_Subgroup_1*		
Acidobacteriota	*Granulicella*		
Bacteroidota	*Flavobacterium*		
Proteobacteria	*Devosia*		
Acidobacteriota	*Bryobacter*		
Proteobacteria	*Brevundimonas*		

Table S11 Co-occurrence network of tea chemical quality components and soil microorganisms

		node	edge	average degree	Average clustering coefficient	average path length
bacteria	April	38	84	4.421	0	3.048
	September	48	155	6.458	0	2.553
fungus	April	36	64	3.556	0	2.864
	September	39	76	3.897	0	2.83

Table S12 Tea chemical quality components and root microorganisms are the core of the network

Bacteria	Fungus
Genus	*Genus*
unclassified_o__Rhizobiales	*Xylogone*
Pseudolabrys	*unclassified_Ascomycota*
norank_c__AD3	*unclassified_o__Helotiales*
norank_o__JG36-TzT-191	*unclassified_f__Hyaloscyphaceae*
norank_f__Micropepsaceae	*Trechispora*
norank_f__Acetobacteraceae	*Saccharomyces*
Mycobacterium	*Mortierella*
Granulicella	*Fusarium*
Chujaibacter	*Aspergillus*
Bradyrhizobium	
Actinospica	

Table S13 PERMANOVA between tea and microbes

	Bacterial				Fungi			
	Apr		Sep		Apr		Sep	
	R^2	*P*	R^2	*P*	R^2	*P*	R^2	*P*
AE	0.288	0.075	0.507	0.007	0.348	0.003	0.548	0.004
Theanine	0.258	0.169	0.59	0.011	0.373	0.004	0.482	0.006
TP	0.243	0.226	0.586	0.014	0.368	0.006	0.543	0.019
Flavone	0.229	0.26	0.581	0.028	0.36	0.007	0.54	0.033
TB	0.229	0.271	0.577	0.028	0.355	0.011	0.527	0.042
EGC	0.235	0.283	0.566	0.031	0.356	0.017	0.522	0.065
EC	0.217	0.311	0.547	0.032	0.36	0.019	0.402	0.067
CAF	0.227	0.328	0.575	0.042	0.341	0.025	0.504	0.071
EGCG	0.216	0.339	0.437	0.049	0.346	0.028	0.399	0.071
ECG	0.215	0.34	0.54	0.075	0.345	0.033	0.49	0.082
GCG	0.226	0.342	0.437	0.079	0.341	0.061	0.514	0.083
C	0.223	0.372	0.507	0.088	0.34	0.079	0.513	0.083
Polysaccharide	0.21	0.379	0.578	0.1	0.284	0.085	0.466	0.089
RM	0.214	0.4	0.546	0.1	0.344	0.1	0.542	0.1
CG	0.207	0.451	0.339	0.19	0.213	0.376	0.239	0.319
GA	0.167	0.713	0.177	0.471	0.186	0.496	0.224	0.4
GC	0.154	0.763	0.127	0.753	0.126	0.889	0.129	0.757

Table S14 Functional microbial screening table

Phylum	Genus	Guild	Confidence	Total
Basidiomycota	*Trechispora*	Wood Saprotroph	Highly Probable	27416.8
Ascomycota	*Penicillium*	Undefined Saprotroph	Possible	13457.2
Ascomycota	*Trichoderma*	Undefined Saprotroph	Possible	11397.8
Basidiomycota	*Auricularia*	Undefined Saprotroph	Probable	5054.2
Ascomycota	*Ilyonectria*	Undefined Saprotroph	Possible	4068.4
Ascomycota	unclassified_o__*Saccharomycetales*	Undefined Saprotroph	Possible	2531.2
Ascomycota	*Trichoderma*	Undefined Saprotroph	Possible	2220.4
Ascomycota	unclassified_f__*Cordycipitaceae*	Undefined Saprotroph	Possible	2096
Ascomycota	*Scutellinia*	Undefined Saprotroph	Probable	1852
Ascomycota	*Trichocladium*	Undefined Saprotroph	Probable	1612.2

Supplementary figures

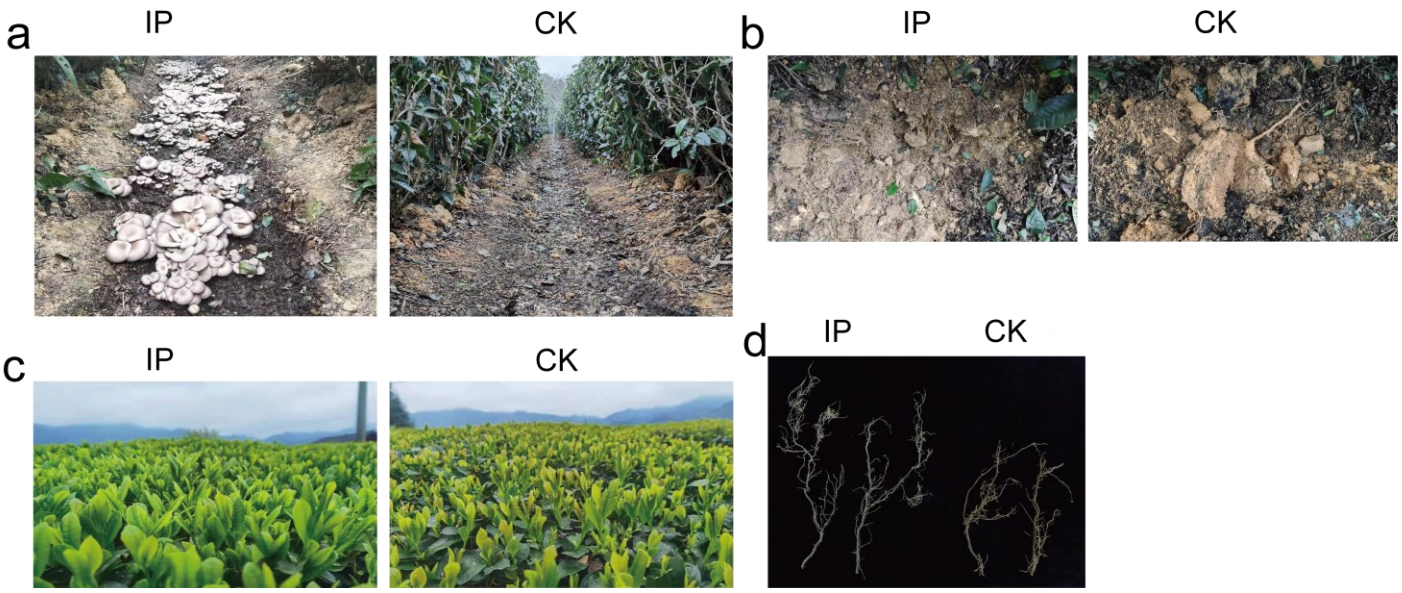

Fig S1 Effects of tea and *Pleurotus ostreatus* intercropping on the growth of tea buds in spring. a is the status of oyster mushroom in the middle of the tea garden, b is the status of tea tree roots in the soil of the tea garden, c is the status of tea tree germination, and d is the status of tea tree root growth. IP is intercropping *P. ostreatus*, CK is treatment, the same below.

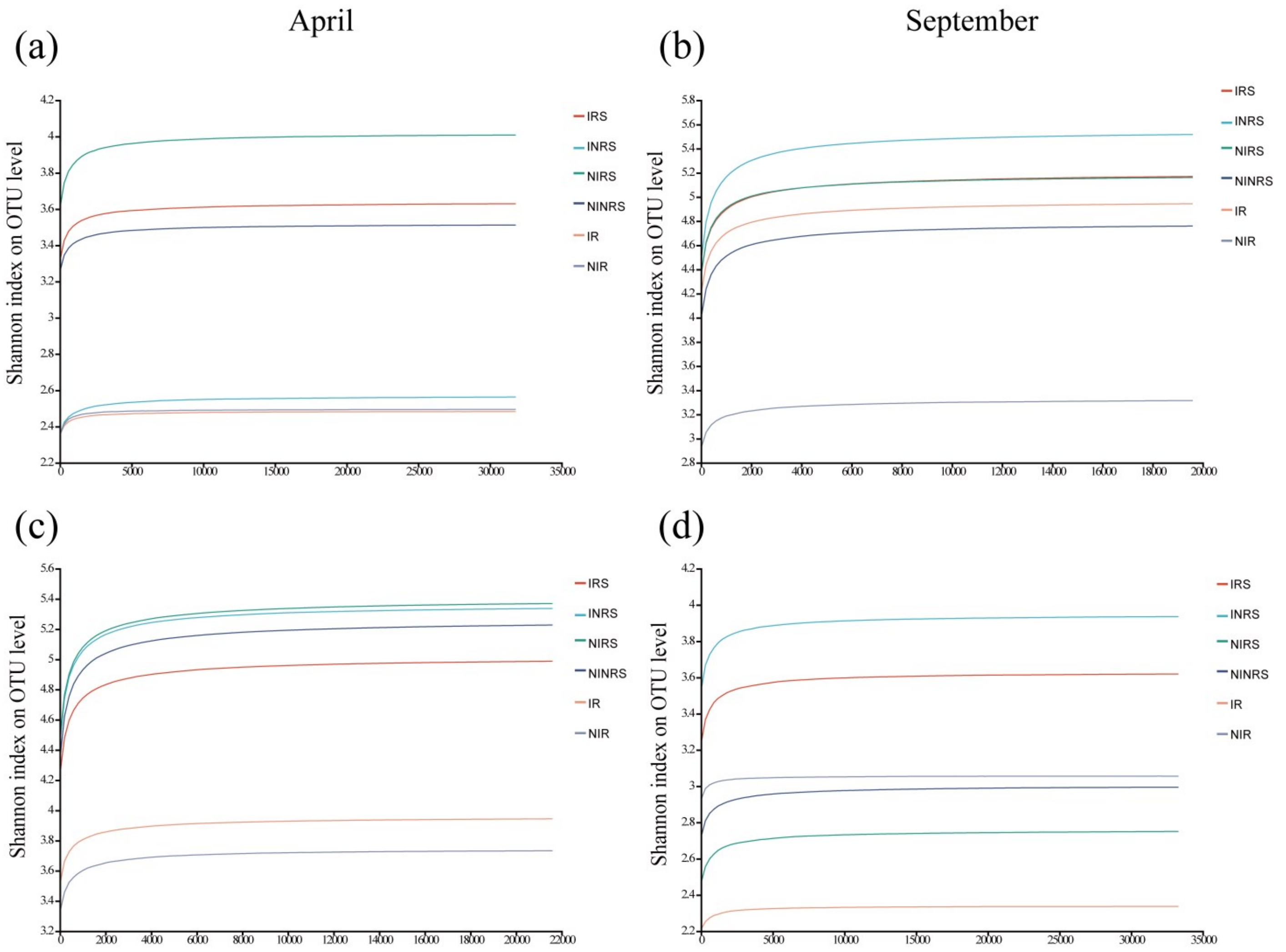

Fig.S2 The Shannon index dilution curve of the microorganisms in the intercropping mushrooms in the tea garden showed that a and b were bacteria, c and d were fungi.

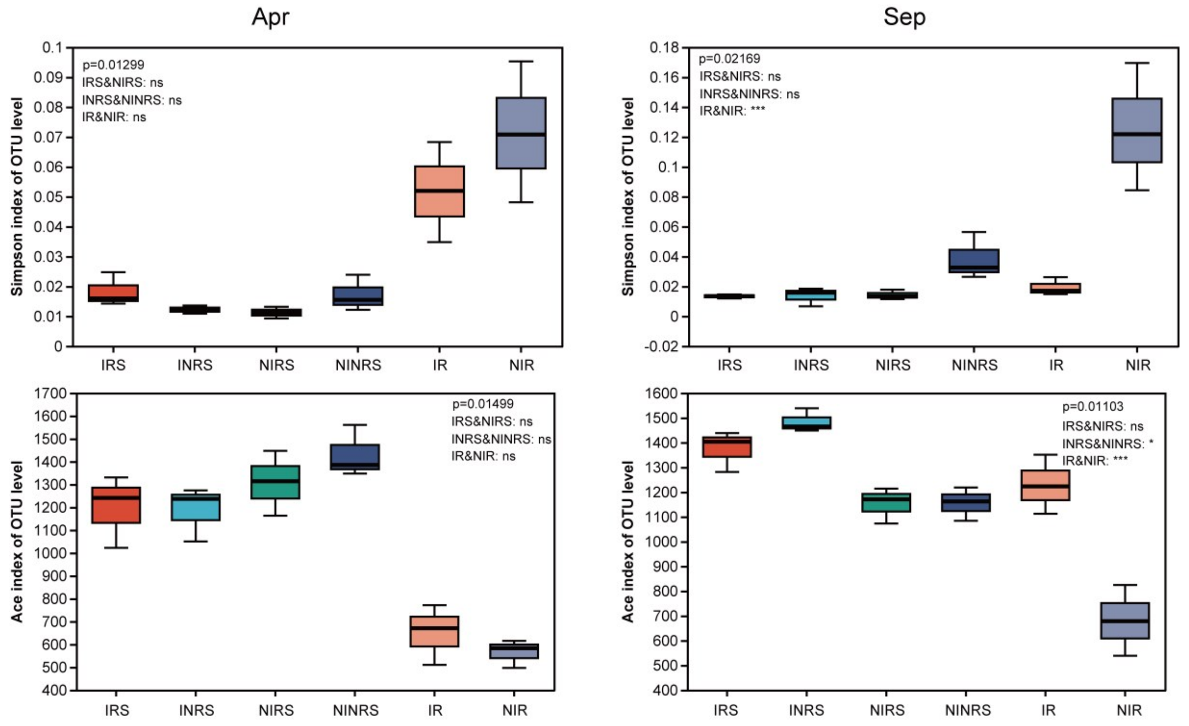

Fig. S3 Ace index and Simpson index of bacteria in different seasons. Apr Represent April, Sep Represent September, the

same below.

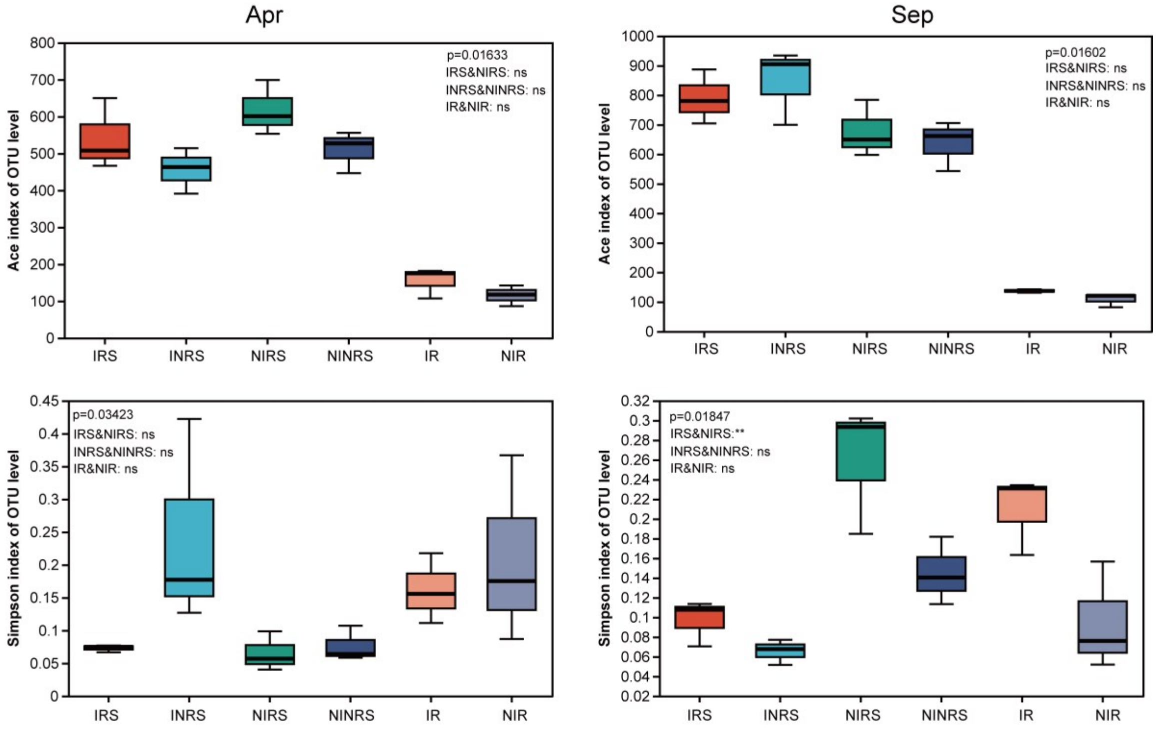

Fig. S4 Ace index and Simpson index of fungi in different seasons.

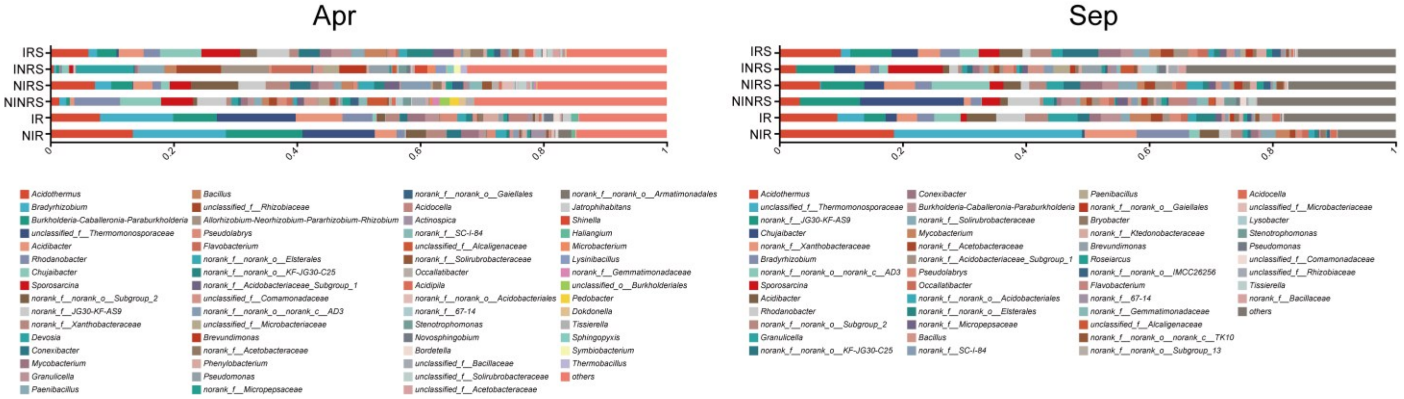

Fig. S5 The relative abundance of *Pleurotus ostreatus* in tea garden intercropping at the level of bacterial genus.

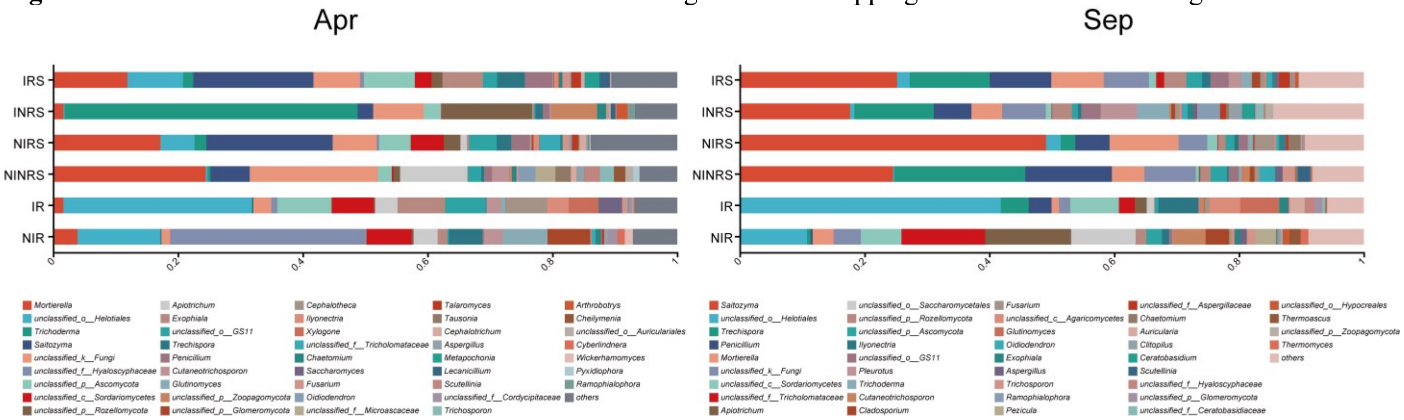

Fig. S6 The relative abundance of *Pleurotus ostreatus* in tea garden intercropping at the level of fungi genus.

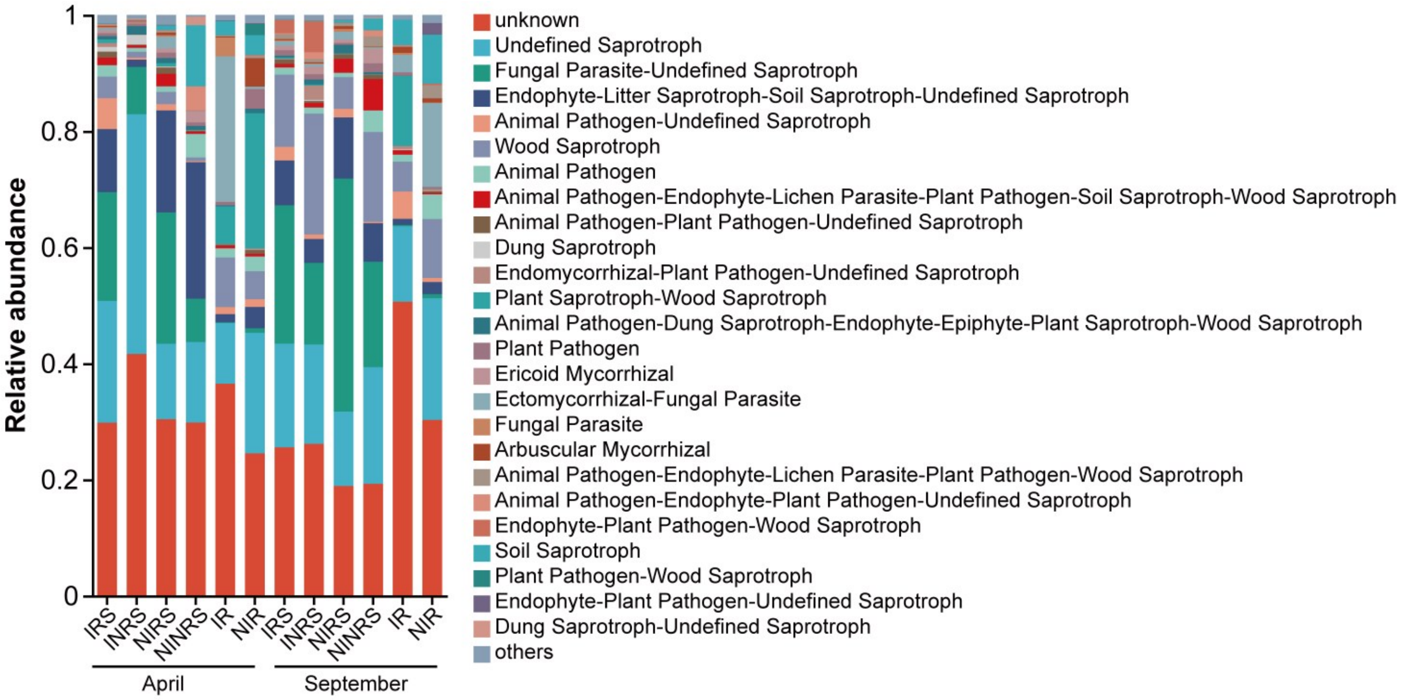

Fig. S7 Variations in the composition of fungal functional groups in tea gardens inferred by FUNGuild.
